# Beyond the usual suspects: emerging uropathogens in the microbiome age

**DOI:** 10.3389/fruro.2023.1212590

**Published:** 2023-07-26

**Authors:** Robert B. Moreland, Brian I. Choi, Wilson Geaman, Caroline Gonzalez, Baylie R. Hochstedler-Kramer, Jerrin John, Jacob Kaindl, Nikita Kesav, Jyoti Lamichhane, Luke Lucio, Malika Saxena, Aditi Sharma, Lana Tinawi, Michael E. Vanek, Catherine Putonti, Linda Brubaker, Alan J. Wolfe

**Affiliations:** ^1^ Department of Microbiology and Immunology, Stritch School of Medicine, Loyola University Chicago, Maywood, IL, United States; ^2^ Bioinformatics Program, Loyola University Chicago, Chicago, IL, United States; ^3^ Department of Biology, Loyola University Chicago, Chicago, IL, United States; ^4^ Department of Obstetrics, Gynecology and Reproductive Sciences, University of California San Diego, La Jolla, CA, United States

**Keywords:** 16S rRNA gene sequencing, anaerobe, antibiotic resistance, facultative anaerobe, metaculturomics, urinary microbiome, urinary tract infection, uropathogen

## Abstract

The advent of sensitive enhanced culture (metaculturomic) and culture-independent DNA-based (metagenomic) methods has revealed a rich collection of microbial species that inhabit the human urinary tract. Known as the urinary microbiome, this community of microbes consists of hundreds of distinct species that range across the entire phylogenetic spectrum. This new knowledge clashes with standard clinical microbiology laboratory methods, established more than 60 years ago, that focus attention on a relatively small subset of universally acknowledged uropathogens. Increasing reports support the hypothesis that this focus is too narrow. Single uropathogen reports are common in women with recurrent urinary tract infection (UTI), although wider disruption of their urinary microbiome is likely. Typical “UTI” symptoms occur in patients with “no growth” reported from standard culture and sometimes antibiotics improve these symptoms. Metaculturomic and metagenomic methods have repeatedly detected fastidious, slow growing, and/or anaerobic microbes that are not detected by the standard test in urine samples of patients with lower urinary tract symptoms. Many of these microbes are also detected in serious non-urinary tract infections, providing evidence that they can be opportunistic pathogens. In this review, we present a set of poorly understood, emerging, and suspected uropathogens. The goal is to stimulate research into the biology of these microbes with a focus on their life as commensals and their transition into pathogens

## Introduction

More than a decade ago, reports began surfacing that challenged the prevailing dogma that urine was typically sterile in the absence of infection (1–10). These studies used high-throughput DNA sequencing (metagenomics) and/or enhanced culture methods (metaculturomics) coupled with matrix-assisted laser desorption/ionization-time of flight (MALDI-TOF) mass spectroscopy (MS) to detect and identify bacteria in urine samples obtained from diverse sets of study participants. These modern sensitive detection methods documented the presence of microbes in urines deemed “no growth” by the traditional or standard urine culture methodologies used by most clinical microbiological laboratories and highlighted the presence of microbes not typically acknowledged as uropathogens (11, 12). These studies and others have resulted in a list of hundreds of taxa. A few taxa are prevalent in individuals without lower urinary tract symptoms. Many more taxa are present in asymptomatic individuals but are more prevalent in those with symptoms (**Table 1**, **Appendix 1**), including those typically associated with urinary tract infection (UTI) and urgency urinary incontinence (UUI), among others (9, 10, 13–21). For a recent review, see (22).

**Table 1:** Frequency of Microbe Identification via Metaculturomics in Patients with and without LUTS^1^.

Microbe	Total N=1007	UTI N=304	UUI N=253	SUI N=50	IC/PBS N=49	Control N=351
*Acinetobacter*	0.50%	0.00%	1.98%	0.00%	0.00%	0.00%
*Actinobaculum*	1.39%	0.99%	4.35%	0.00%	0.00%	0.00%
*Actinomyces* ^2^	10.13%	7.57%	22.53%	4.00%	6.12%	4.84%
*Actinotignum*	5.26%	3.95%	13.04%	6.00%	0.00%	1.42%
*Aerococcus*	16.29%	13.49%	36.36%	16.00%	10.20%	5.13%
*Aerococcus urinae*	14.20%	11.18%	33.60%	14.00%	8.16%	3.70%
*Alloscardovia omnicolens*	6.65%	4.93%	13.83%	8.00%	4.08%	3.13%
*Bacillus*	0.40%	0.33%	0.40%	0.00%	0.00%	0.57%
*Bifidobacterium*	5.06%	4.28%	9.88%	0.00%	8.16%	2.56%
*Brevibacterium*	3.28%	2.30%	8.70%	2.00%	2.04%	0.57%
*Candida*	3.18%	1.97%	7.11%	4.00%	2.04%	1.42%
*Citrobacter*	0.60%	1.64%	0.40%	0.00%	0.00%	0.00%
*Corynebacterium*	22.34%	15.13%	51.78%	26.00%	16.33%	7.69%
*Cutibacterium*	1.19%	0.99%	2.77%	0.00%	4.08%	0.00%
*Dermabacter hominis*	0.79%	0.33%	2.37%	2.00%	0.00%	0.00%
*Enterobacter*	1.79%	0.33%	5.53%	0.00%	0.00%	0.85%
*Enterococcus*	11.42%	8.88%	23.72%	8.00%	8.16%	5.70%
*Enterococcus faecalis*	11.12%	8.88%	22.92%	8.00%	8.16%	5.41%
*Escherichia coli*	24.83%	50.99%	25.69%	12.00%	8.16%	5.70%
*Facklamia hominis*	4.07%	1.32%	12.25%	2.00%	0.00%	1.42%
*Gardnerella*	14.10%	11.84%	20.95%	12.00%	2.04%	13.11%
*Gemella*	0.40%	0.00%	1.58%	0.00%	0.00%	0.00%
*Globicatella*	0.50%	0.00%	1.98%	0.00%	0.00%	0.00%
*Haematomicrobium*	0.30%	0.99%	0.00%	0.00%	0.00%	0.00%
*Haemophilus*	0.89%	0.33%	1.58%	4.00%	2.04%	0.28%
*Klebsiella*	5.76%	11.51%	6.72%	0.00%	2.04%	1.42%
*Klebsiella pneumoniae*	4.57%	8.22%	5.93%	0.00%	2.04%	1.42%
*Kocuria*	0.30%	0.00%	0.79%	0.00%	2.04%	0.00%
*Lactobacillus*	37.24%	35.53%	56.13%	42.00%	30.61%	26.21%
*Micrococcus*	3.38%	0.99%	7.11%	0.00%	2.04%	3.42%
*Moraxella*	0.30%	0.00%	0.79%	0.00%	0.00%	0.28%
*Morganella*	0.50%	0.33%	1.19%	0.00%	2.04%	0.00%
*Neisseria*	0.89%	0.66%	1.19%	6.00%	0.00%	0.28%
*Oligella*	1.19%	0.66%	3.56%	2.00%	0.00%	0.00%
*Peptoniphilus*	0.50%	0.00%	1.98%	0.00%	0.00%	0.00%
*Prevotella*	0.30%	0.00%	0.79%	2.00%	0.00%	0.00%
*Proteus*	2.38%	4.28%	3.95%	0.00%	0.00%	0.28%
*Pseudoglutamicibacter*	3.67%	1.64%	10.67%	0.00%	4.08%	0.85%
*Pseudomonas aeruginosa*	1.29%	1.97%	2.77%	0.00%	0.00%	0.00%
*Rothia*	1.79%	0.66%	2.77%	10.00%	0.00%	1.14%
*Staphylococcus*	22.44%	16.45%	45.85%	30.00%	20.41%	9.97%
*Coagulase Negative Staphylococcus*	21.05%	14.47%	45.06%	24.00%	20.41%	5.41%
*Coagulase Positive Staphylococcus* ^3^	2.38%	2.30%	3.56%	8.00%	0.00%	0.28%
*Streptococcus*	36.35%	28.29%	63.64%	40.00%	28.57%	24.22%
*Streptococcus viridans grp.*	13.90%	7.89%	22.92%	14.00%	14.29%	4.56%
*Streptococcus anginosus grp.*	23.24%	16.45%	48.62%	26.00%	12.24%	6.84%
*Streptococcus agalactae*	8.04%	7.89%	11.86%	10.00%	10.20%	4.84%
*Trueperella bernardiae*	2.09%	0.99%	5.53%	4.00%	0.00%	0.57%
*Winkia neuii*	9.43%	7.24%	21.74%	10.00%	4.08%	3.13%
***Unknown***	24.03%	17.76%	57.71%	18.00%	2.04%	9.12%
*Arthrobacter*	0.10%	0.00%	0.40%	0.00%	0.00%	0.00%
*Aureimonas*	0.10%	0.00%	0.40%	0.00%	0.00%	0.00%
*Bacteroides*	0.10%	0.00%	0.40%	0.00%	0.00%	0.00%
*Blastocystis*	0.10%	0.33%	0.00%	0.00%	0.00%	0.00%
*Brevundimonas*	0.10%	0.00%	0.00%	0.00%	0.00%	0.28%
*Campylobacter*	0.20%	0.00%	0.40%	0.00%	0.00%	0.28%
*Comamonas*	0.10%	0.00%	0.40%	0.00%	0.00%	0.00%
*Dialister*	0.20%	0.00%	0.40%	0.00%	0.00%	0.28%
*Dolosigranulum*	0.10%	0.33%	0.00%	0.00%	0.00%	0.00%
*Eikenella*	0.10%	0.00%	0.40%	0.00%	0.00%	0.00%
*Finegoldia*	0.20%	0.00%	0.40%	0.00%	2.04%	0.00%
*Fusobacterium*	0.10%	0.00%	0.40%	0.00%	0.00%	0.00%
*Kytococcus*	0.10%	0.33%	0.00%	0.00%	0.00%	0.00%
*Propionimicrobium*	0.20%	0.00%	0.79%	0.00%	0.00%	0.00%
*Rhizobium*	0.20%	0.00%	0.79%	0.00%	0.00%	0.00%
*Saccharomyces*	0.10%	0.00%	0.40%	0.00%	0.00%	0.00%
*Serratia*	0.10%	0.33%	0.00%	0.00%	0.00%	0.00%
*Slackia*	0.10%	0.00%	0.40%	0.00%	0.00%	0.00%
*Stenotrophomonas*	0.10%	0.00%	0.40%	0.00%	0.00%	0.00%
*Veillonella*	0.20%	0.00%	0.79%	0.00%	0.00%	0.00%
*Weeksella*	0.20%	0.00%	0.79%	0.00%	0.00%	0.00%

1 Isolates were isolated by EQUC and Identified via MALDI-TOF mass spectrometry. Frequency was calculated by dividing the total count of isolations of each genus/species/group by the total number of samples in each group (N). Patients can be colonized by more than one species/genus at a time. When calculating frequency, redundancies in genus/group were considered. The ‘Unknown’ grouping represents isolates unidentifiable via MALDI-TOF MS.

2 The values for the genus Actinomyces includes members of the newly reclassified genera Gleimia and Schaalia, as well true Actinomyces species.

3 The values for Coagulase-positive Staphylococcus are almost all S. aureus.

## Statement of purpose

The purpose of this review is to highlight a set of poorly understood, emerging, and suspected uropathogens. The intent is to generate momentum for prospective and retrospective studies to identify risk factors and improve antibiotic surveillance, especially for those species that have no Clinical and Laboratory Standards Institute (CLSI) standards. We also wish to encourage investigations into the pathophysiology of these microbes. Thus, with a few exceptions, this review will focus on these lesser-known microbes, including members of the families *Aerococcaceae*, *Actinomycetaceae*, and *Bifidobacteriaceae.* Also discussed will be members of the *Streptococcus anginosus* group (SAG) and *Enterococcus faecalis*. While SAG members have long been considered to be commensals, increasing evidence supports the conclusion that they are more likely opportunistic pathogens (23). Although long accepted as a pathogen, the comparatively well-studied *E. faecalis* has become increasingly implicated in urinary tract disorders and its pathophysiology within the urinary tract remains understudied (24).

Other species we will review are anaerobes, specifically members of the orders *Eubacteriales* and *Bacteroidales.* Traditionally, anaerobes have not been considered to be uropathogenic (25, 26). However, in this age of metagenomics, metaculturomics, and MALDI-TOF identification, this dogma is being reexamined (27). The concept that oxygen is toxic to obligate anaerobes (28) does not account for the strategies these microbes use to survive and flourish in human organ niches (29, 30), including the urinary tract.

Of organisms reviewed here, some are aerobes, some are facultative anaerobes, and some are strict anaerobes. Many are fastidious. As such, classical clinical laboratory diagnosis using standard urine culture (SUC) methods would not detect most of these potential uropathogens in the time frame or atmospheric conditions of the assay (12, 31). In contrast, all taxa reviewed herein have been detected by metagenomic approaches, including 16S rRNA gene sequencing and shotgun metagenomic sequencing (9, 10, 14, 15, 18, 19, 32–34), and/or metaculturomic methods, such as Expanded Quantitative Urine Culture (8, 12, 21) (**Table 1**). Despite the discovery of some of these microbes as much as a century ago, little is known about their biology.

Since much has been written about the most commonly accepted and best studied uropathogens, including members of the family *Enterobacteriaceae* (e.g., the genera *Escherichia, Klebsiella, Enterobacter*, and *Proteus*), and Gram-negative saprophytes (*Pseudomonas aeruginosa* and *Acinetobacter baumannii*), we will discuss them only briefly (35). The same is true for the better-known member(s) of the streptococci (*S. agalactiae*), the coagulase-positive staphylococi (*S. aureus*), and the coagulase-negative staphyococci (*S. saprophyticus, S. epidermidis*, and *S. haemolyticus*), as well as the yeast genus *Candida (C. albicans*). We will not review the best-known anaerobes, including but not limited to the genera *Porphyromonas, Sneathia*, and *Peptoniphilus.* Finally, some urinary microbes have no cell wall, most notably *Mycoplasma* and *Ureaplasma* (36). While specialized techniques exist for the culture of these microbes (27, 37, 38) and rapid molecular diagnoses have been reported for both (39), we will not review these organisms here. For recent reviews on the taxa mentioned above, see (24, 40–46).

## Commensals versus pathogens

The standard approach to treating UTI is based on Koch’s postulates, which assumes a single organism is responsible for pathogenicity, that this organism can be isolated from the diseased tissue/fluid, be able to reproduce the disease state in a healthy experimental system and be recovered afterward in pure culture (47). This highly successful approach was responsible for the elucidation of the bacterial pathogens of nineteen different diseases from 1877 to 1906 including anthrax, bubonic plague, cholera, diphtheria, pediatric diarrhea, bacterial pneumonia, gonorrhea, syphilis, tuberculosis, typhoid fever, and whooping cough (47). However, the discovery of the human microbiome and existence of eubiotic states within human tissues such as the dermis, and the respiratory, gastrointestinal, and urogenital tracts caused a rethinking of the roles played by bacteria in health and disease (48, 49).

An initial cataloging of human urinary bladder isolates revealed 149 distinct species ranging from aerobes and microaerobes to facultative anaerobes and anaerobes (50). While all these microbes can be identified by DNA-dependent methods, most are not culturable or grow poorly under standard urine culture conditions, while others overgrow because they possess adaptive advantages (12). This is particularly true for facultative anaerobes and is consistent with the genera *Escherichia*, *Pseudomonas*, *Klebsiella, Proteus, Staphylococcus*, and *Enterococcus* being among “The Usual Suspects” and common to standard urine culture diagnoses (29, 51). Microbes within the microbiome can be grouped into six different classes: non-pathogen (not causing disease), a pathogen (causing disease), a commensal (tissue resident, benefiting the host) a symbiont (tissue resident, benefiting the host and is benefited from the host), a colonizer (tissue resident and may or may not be disease causing) and a pathobiont (tissue resident, generally beneficial but can cause disease under special conditions) (52).

To understand microbial communities, one must first isolate and characterize each of the individual species. Establishing the commensal status of a species is much more difficult than reporting pathogen case reports in tissues. Consequently, many of the reports in the literature regarding the species reviewed here are pathological reports from abscesses, blood cultures, or other disease states. The science of understanding the interactions in a microbial community between the six types of microbes mentioned above is in its infancy (52, 53). While reports of infections in tissues other than the urinary tract provide only a worst-case capability of the capacity of these species to cause or contribute to disease, it emphasizes the major thrust of this review - to study these species in context and to begin to understand their interaction within communities.

## Commonly accepted uropathogens

Approximately 150 million people worldwide are diagnosed each year with UTIs (54). These infections are thought to be caused by uropathogenic bacteria, including but not limited to members of the genera *Escherichia*, *Pseudomonas*, *Klebsiella, Proteus, Staphylococcus*, and *Enterococcus* (51 *).* Note that many of these species are in the World Health Organization’s ESKAPE list of critical pathogens (55). *Escherichia coli* is considered to be the most common cause of UTIs. Other bacterial species that are commonly associated with UTI-like symptoms include *Pseudomonas aeruginosa, Klebsiella pneumoniae, K. oxytoca, Enterococcus faecalis, E. faecium, Proteus mirabilis, Proteus vulgaris, Staphylococcus aureus*, and *S. saprophyticus.* The yeast species *Candida albicans* also can cause UTI-like symptoms (56, 57). For example, a study of 727 hospitalized urological patients diagnosed with nosocomial acquired UTI reported the most commonly pathogens detected by SUC to be *E. coli* (31%), followed by species of the genera *Pseudomonas* (13%), *Enterococcus* (10%), *Klebsiella* (10%), *Enterobacter* (6%) and *Proteus* (6%) (58). These taxa are all fast growing, non-fastidious, and able to thrive in the presence of ambient oxygen (PO_2_ 150mmHg, 20kPa) (29, 59), characteristics that facilitate detection by SUC (60). Other microbes that are easily detected by SUC include additional members of the Gram-negative family *Enterobacteriaceae*, such as the genera *Serratia, Citrobacter, Morganella, Providencia*, and *Pantoea*. All have the capacity to be pathogenic, but these genera are detected quite rarely. For example, in a re-examination of several of our previous studies (21), they were each detected in the catheterized (bladder) urine of less than 0.1% of adult females (n=1007) and were rare even in those with UTI-like symptoms (**Table 1**).

### Saprophytes and other environmental pathogens

Saprophytes are organisms that obtain their nutrients from decaying organic material. As such, they tend not to be obligate infectious agents of humans. However, they can be opportunistic pathogens, causing wound and nosocomial infections, primarily in immunocompromised individuals. A recent systematic review found saprophytic bacteria to be implicated in hundreds of infections in dozens of countries (42); 5% were UTIs. Most affected individuals had comorbidities and the most common species detected were *Pantoea aglomerans*, *Klebsiella (formerly Enterobacter) aerogenes*, and *Pseudomonas putida*. The authors warn that saprophytes such as these may become more common in healthcare settings like other opportunistic environmental Gram-negative bacteria, especially *Acinetobacter baumannii* and *P. aeruginosa* (42).

*A. baumannii* and *P. aeruginosa* may cause nosocomial infections, including nosocomial-acquired UTIs, especially in frail or immunocompromised individuals (61). The World Health Organization considers both priority-1 (critical) pathogens because of their tendency to be resistant to carbapenems and third generation cephalosporins, which are considered to be last resort antibiotics (55, 62). Multi-drug resistance and their biofilm-forming capacity makes these infections difficult to treat with antibiotic therapy (63). Whereas efforts to understand *P. aeruginosa* and *A. baumannii* pathophysiology have been extensive, uropathogenic strains remain understudied (42, 63–67).

### Fungi

Fungal UTIs are generally caused by members of the genus *Candida* (68). Of these, the best known and most common UTI-associated species is *C. albicans*. Other species include *C. glabrata, C. parapsilosis*, and *C. auris.* The latter is an emerging pathogen associated with UTIs that the CDC has added to its surveillance list because it tends to be multidrug resistant, is difficult to detect using standard clinical laboratory methodology, and has caused multiple outbreaks in healthcare settings (69–71). Diabetes, catheterization, hospitalization, and broad-spectrum antibiotics are risk factors for *Candida* infections (72). Azole antifungals are the most common treatment for symptomatic infections; however, increasing resistance has been observed in clinical isolates. Wider surveillance studies are severely needed (73).

The diagnostic criteria for detecting *Candida* in urine samples are not standardized with continuing debate about reporting thresholds (74, 75). More problematically, typical clinical laboratory methods of detection have poor sensitivity for *Candida* species, even *C. albicans*. Several prospective studies that cultured urine on the standard fungal medium, Sabouraud dextrose agar, have reported greater numbers of non-*C. albicans* species than standard urine culture methods (75–77). Thus, *Candida* species are often not detected by standard clinical laboratory testing and consequently are underreported.

### The genus *Staphylococcus*

The genus *Staphylococcus* is comprised of more than 40 species of Gram-positive, facultative anaerobic cocci (78–80). From a clinical microbiological diagnostic point, the genus can be divided by coagulase activity (conversion of fibrinogen to fibrin). The most common coagulase-positive *Staphylococcus* is *S. aureus*, a commensal skin and upper respiratory tract coccus known to be a potent, antibiotic-resistant opportunistic pathogen that can cause diverse infections, especially skin and soft tissue infections and toxic shock syndrome (80, 81). As such, surveillance for this uropathogen is high. In our re-examination of isolates obtained from catheterized bladder urine samples of ~1000 adult females (21), *S. aureus*, the most common coagulase-positive *Staphylococcus*, was detected in urine but it was not prevalent (**Table 1**).

In contrast, coagulase-negative staphylococci (CoNS) are often dismissed as contaminants (80, 82–84). As opportunistic pathogens in the urinary tract, CoNS are associated with UTIs, uncomplicated, catheter-associated, and nosocomial (82, 85–88). The ability for this genus to acquire antibiotic resistance makes this group of microbes an increasing threat to infectious disease control (81). We found them to be quite prevalent, especially in adult females diagnosed with UUI (**Table 1**). Of the 11 CoNS species detected, here we review the 2 most prevalent species and 1 species commonly associated with UTI (*S. epidermidis*, *S. haemolyticus* and *S. saprophyticus*, respectively).

*S. saprophyticus* was first recognized as a causative microbe for UTI in young females (82, 86), and does appear to be associated with young females of reproductive age. In contrast, it appears to be very rare in older females; we have never detected it in this population. However, the susceptibility of the host by age and reproductive status remains unclear. Virulence factors, including urease activity, have been described (82, 83, 89).

The most common CoNS in the urinary tract is *S. epidermidis* (41, 87). Whereas *S. epidermidis* infections are rarely life-threatening, increasing antibiotic resistance and biofilm-forming ability make them difficult to treat with antibiotics. Investigations into the underlying molecular mechanisms have been performed (87). Numerous case reports implicate *S. epidermidis* in UTIs, especially in children (90–92), but the pathophysiology of urinary isolates has yet to be explored.

*S. haemolyticus* is the second-most isolated CoNS from urine. It is also common in blood cultures, especially from immunocompromised patients. As such, it is considered an emerging multidrug-resistant nosocomial pathogen (83, 84). Of particular concern is the ability of *S. haemolyticus* to acquire multiple antibiotic resistance genes, making antibiotic stewardship in the global treatment of UTIs an urgent public health issue (84, 93). The incidence of *S. haemolyticus* UTIs are increasingly reported (84, 88).

## Emerging uropathogens

In contrast to several of the universally acknowledged uropathogens, including but not limited to *Serratia, Morganella, Citrobacter*, and *E. faecium*, many emerging or suspected uropathogens are considerably more prevalent (**Table 1**). They have been underappreciated for 2 major reasons. First, as mentioned above, many simply do not grow or grow poorly under SUC conditions (60); however, they can be grown (**Appendix 2** **)**. Even *E. faecalis* tends to be underreported, in part due to overgrowth of faster growing species (31). Second, before the advent of MALDI-TOF MS, accurate identification of many species was difficult (94) and many would have been dismissed as contaminants (95–97). This dismissal has its consequences as was suggested by investigators studying polymicrobial infections in urinary sepsis where contamination could be ruled out (98, 99). The growth of microbes at less than 10^5^ colony forming units per milliliter (cfu/mL) has been noted and its significance debated since the initial report of this standard for “infection” (100, 101). On the opposite end of the spectrum and demonstrating that some microbes that do not grow on SUC, negative standard urine cultures was reported in women with lower urinary tract symptoms; with treatment, negative cultures and the symptoms persisted (102), implying that some other causal factor and/or uncultivated microbe was present.

Because its role in lower urinary tract health has been underappreciated, we will review *E. faecalis* first and then a set of emerging and suspected uropathogens.

### The species *Enterococcus faecalis*

Less than 30 years after being recognized as a distinct taxon, the clinical outlook on *Enterococcus* transitioned from harmless gut commensal to a major public health concern. *E. faecalis*, the most common clinical enterococcal species, is ubiquitously present in the human gut microbiome where it plays a crucial role in nutrient metabolism and maintenance of a heathy gut environment (103, 104). However, these microbes are also adept at adapting to novel environments and transferring DNA to members of its own genus, as well as other taxa. This latter characteristic has greatly contributed to the worldwide spread of antibiotic resistance, the most notable being the cassette of genes responsible for vancomycin resistance, which is attributed to significantly increased mortality rates (105).

With or without antibiotic resistance genes, enterococcal infections at many body sites exhibit increased risk of persistence and recurrence in comparison to other common pathogens (106). Mechanisms underlying these chronic infection phenotypes are largely unknown, as previous comparative phylogenomic studies have been unable to differentiate between clinical isolates from diverse infection types (107), most likely due to insufficient isolate numbers and metadata. Despite this, *E. faecalis* epidemiology and pathogenesis are most often studied in the context of nosocomial infections. These investigations have elucidated the presence and putative function of various virulence factors, including proteins that facilitate colonization, aggregation, and toxin production (107). The most severe enterococcal nosocomial infection is bacteremia, which can lead to sepsis and endocarditis. Even with appropriate treatment, this infection is fatal in nearly 30% of cases (108). Enterococcal bacteremia has previously been thought to originate *via* fecal contamination of venous catheters or other medical devices; however, recent studies have identified ascending bladder infections as a frequent prelude to bacteremia (103, 109).

Patients with long-term indwelling urinary catheters have increased risk for enterococcal bacteremia and sepsis. Therefore, catheter-associated UTI (CAUTI), the most common enterococcal nosocomial infection, is a main model system used to assess *E. faecalis* behavior in the bladder. Studies have shown that *E. faecalis* acts as a “founder species” in catheter colonization and that *E. faecalis* presence in polymicrobial infections increases virulence of other uropathogenic microbes, including *P. mirabilis* and *E. coli* (110, 111). CAUTI is thought to result from fecal contamination of indwelling urinary catheters (103); however, the discovery of the bladder microbiome raises the possibility that the bladder and urethra could serve as endogenous reservoirs for *E. faecalis*, making it possible that community-acquired UTI and subsequent persistent bladder colonization could precede these chronic/recurrent infection phenotypes.

Although *E. faecalis* is a recognized uropathogen underlying community-acquired UTI, SUC has a detection rate of only 50% relative to EQUC (31). This is because *E. faecalis* is often cultured alongside other uropathogens or commensals, meaning it is either (1) outcompeted during culture by hardier organisms, such as *E. coli*, or (2) dismissed as “mixed morphologies” and reported as contamination. Missed detection and empiric treatment of *E. faecalis*-UTI imparts considerable risk, as the efficacy of many antibiotics commonly used to treat UTI is currently being debated for this species. The adaptability of this species and its ability to acquire antibiotic resistance even to the newest antibiotics has correlated with an increased number of cases reported and represents a substantial health risk (24, 35, 111). These include resistance to aminoglycosides (including gentamycin and kanamycin), β-lactams, chloramphenicol, clindamycin, daptomycin, erythromycin, flouroquinolones, oxazolidinones, rifampin, streptomycin, tetracyclines, and tigecycline (24).

This is extremely problematic, as this species is known to have a tropism for kidneys and once ascended is difficult to eradicate (103, 112). Recently, *E. faecalis* has also been associated with populations experiencing recurrent UTI (31, 113, 114), defined as 3+ UTI in a year or 2 within 6 months (115). These data suggest that *E. faecalis* behavior in the bladder mimics that of common nosocomial infections, strengthening the concern that this species could be responsible for more severe infection phenotypes.

Thus far, no studies have reported how *E. faecalis* alters the host bladder environment to promote its own persistent colonization. Additionally, no studies have identified the virulence genes necessary for persistent bladder colonization or urothelial cell invasion. Understanding enterococcal behavior, especially in connection to recurrent UTI, is crucial for developing more efficacious treatment and prevention of severe infections, such as bacteremia and sepsis.

### The family *Aerococcaceae*

Understudied and under-detected, members of the family *Aerococcaceae* are easily mistaken for other Gram-positive cocci with similar morphologies and strict growth requirements. Their taxonomy and identification have been fraught with inconsistency and their relationship with human disease is frustratingly mysterious (116). The increasing isolation of these organisms from the urine of sick humans has earned them the title of emerging uropathogens (116). Indeed, their ability to cause serious disease, such as infectious endocarditis, makes them a clear threat, and yet their ability to cause disease is still uncharacterized. Below, we consider the genera *Aerococcus*, *Facklamia*, and *Globicatella.*

#### Aerococcus.

The genus *Aerococcus* consists of several species, the majority of which are associated with the urogenital tracts of livestock and humans. The most prevalent and threatening species, however, is *Aerococcus urinae.* **Table 1** shows enrichment in adult females diagnosed with UTI or UUI relative to asymptomatic controls (detected in 11%, 34%, and 4%, respectively). Thus, *A. urinae* is implicated with urine, especially in adult females with LUTS. However, the circumstances and implications of how it ends up there remains a mystery. The natural reservoir of the bacterium is poorly described and the circumstances in which it becomes pathogenic are uncharacterized. Currently, there is a demonstrative need for greater investigation into the involvement of *A. urinae* in urinary tract disorders such as UTI and UUI, as well as invasive tissue infections. With increasing antibiotic resistance observed in clinical isolates, *A. urinae* poses a growing threat to the undiagnosed (and misdiagnosed) patient.

The first isolates of *A. urinae* came from the urine of patients diagnosed with UTI (117). Originally thought of as a rare cause of human infection, the bacterium has since seen a clear rise in diagnoses and case reports alongside improvements in culture techniques and identification technologies (118–121). While lethal cases are rare, *A. urinae* has been identified in a variety of severe disease complications, such as soft tissue infections and bacteremia, all traced to a urological origin (120, 122–124). Non-invasive infections are associated with UTI and UUI in women (**Table 1**) (9, 21). However, enhanced culturing of urine from asymptomatic participants also detects this species, complicating characterization of *A. urinae’s* status, and suggesting that it is an opportunistic pathogen (125).

Monoculture of *A. urinae* from urine is uncommon; instead, it is often identified alongside several other species, contributing to its dismissal as a contaminant. In cases of bacteremia, however, the majority of infections are monomicrobial with significant risk for endocarditis and septic embolization (126, 127). Thus, it remains unclear whether this bacterium works in concert with others or on its own.

Risk factors for invasive infections include older age and comorbid genitourinary diseases (121, 124, 128). In pediatric settings, *A. urinae* has been reported as a cause for extraordinary malodorous urine in boys with comorbid urogenital disorders as a risk factor (129, 130). Malodorous urine has been documented in adult patients as well, having been described as ammoniacal and “socially disabling” (120, 131).

In all severe cases of infection, misidentification and lack of resistance testing can lead to fatality (132, 133). Currently, the criterion standard for rapid identification in the clinical setting is *via* MALDI-TOF MS (125). However, *A. urinae* is easily missed on routine urine culture and other bacteriological tests and, when isolated, is often misidentified as streptococci, staphylococci, or enterococci because they share many characteristics.

Whole genome sequencing and phenotypic characterization of the organism has revealed substantial diversity within the *A. urinae* species designation such that subdivision has been suggested (134–136), although the clinical relevance of such divisions remains unknown. Like other invasive uropathogens, *A. urinae* demonstrates the ability to form biofilms on catheters and heart tissue, as well as the ability to aggregate platelets (137–139). The first UTI mouse model for *A. urinae* demonstrated a tropism for the kidney, indicating a route for ascending infection despite the bacterium being non-motile (140). Analysis for virulence factors revealed genes predicted to be associated with adhesion and anti-phagocytosis (135). Proteomic studies have supported this finding, revealing an abundance of adhesive surface proteins expressed on the bacterium’s surface (138, 141). Unfortunately, no genetic model currently exists to allow mechanistic studies into these virulence factors.

With proper identification and susceptibility testing, antibiotic therapy is generally effective for *A. urinae* infection. Isolates from several studies have demonstrated susceptibilities to most antibiotics used against Gram-positive organisms; however, resistances have been indicated to fluoroquinolones, cephalosporins, trimethoprim-sulfamethoxazole, and tetracycline (142–146). There is concern that antibiotic resistance may be increasing; rising resistance has been detected in wastewater samples (147). As with the related streptococci, staphylococci, and enterococci, the possibility of horizontal gene transfer of resistance genes may pose a significant future risk. Another member of this genus, *A. urinaeequi*, has been found to harbor a plasmid with tetracycline resistance and a transposable element with vancomycin resistance (148, 149). As such, prudent stewardship based on careful microbial identification is foundational for the diagnosis and treatment of *A. urinae* infections.

#### Facklamia.

*Facklamia* species are challenging to accurately identify with current microbiologic systems; they are often confused with hemolytic streptococci (150). Thus, *F. hominis* is an underrecognized pathogen that has been isolated from a variety of clinical specimens, including bacteremia associated with brain and soft tissue abscesses, endocarditis, necrotizing gangrene, and ischemic stroke symptoms (151). It also has been associated with pediatric pyelonephritis (152), acute cystitis and urosepsis (152), as well as bacteremia associated with transurethral resection of the prostate (153). Despite isolation from vaginal specimens and urine, especially in adult females with UUI (**Table 1**), the role of *F. hominis* as a commensal and the transition to opportunistic pathogen has yet to be explored (151). Antibiotic resistances have been demonstrated towards cephalosporins, erythromycin, clindamycin, and trimethoprim-sulfamethoxazole (150, 151). More studies are needed to investigate mechanisms of virulence, predisposing risk factors, and rates of infection.

#### Globicatella.

*G. sanguinis* was first isolated from human blood in 1978; more recently it was proposed to be its own novel genus (154). *Globicatella* infections have been associated with bacteremia, septicemia, meningitis, infective endocarditis, wound infections, and UTIs in humans on a sporadic basis (154–156). Isolates have been detected in catheter-associated biofilms along with *A. urinae* (138). As such, *G. sanguinis* is now considered to be an emerging pathogen with an expanding disease spectrum, recently identified from patients with endophthalmitis and osteomyelitis (157, 158). Since this species also has been considered to be a commensal bacterium (159), it likely should be considered an opportunistic uropathogen. Because of its close resemblance to streptococci and aerococci under microscopic examination and morphologically on blood agar, *G. sanguinis* is often misidentified. As a result, it can be easily underestimated in clinical settings (160).

### The *Streptococcus anginosus* (*Streptococcus milleri*) group

The Gram-positive coccus *Streptococcus anginosus* was originally described in 1906 (161). Early on, *S. anginosus* was thought to cause strep throat, as it was observed to induce inflammation of the fauces, the arched opening at the back of the mouth that leads to the pharynx (161–163). The high degree of heterogeneity in phenotypic characteristics between strains of *S. anginosus* (161, 164, 165) led to conflicting taxonomic characterizations during early studies before Whiley and Beighton disambiguated *S. anginosus* into three separate species: *S. intermedius*, *S. constellatus*, and *S. anginosus*. Together, these species comprise the *Streptococcus anginosus* Group (SAG), also known as the *Streptococcus milleri* group, which is one group within the larger set of viridans streptococci (162). Today, it is well known that all three members of SAG are part of the normal human flora, having been isolated from the oropharynx, gastrointestinal tract, and vagina of healthy individuals (23, 162–164, 166). As such, they are generally not considered pathogens. However, in immunocompromised individuals, opportunistic infections leading to bacteremia, pharyngitis, and purulent infections have been reported (23). SAG also may contribute to pulmonary exacerbations in cystic fibrosis patients (167, 168). They also contribute to cases of infective endocarditis (169, 170), and have been reported as complications of otitis media and sinusitis and intracranial infections in children (171, 172).

While SAG is primarily isolated from the upper respiratory tract, an increasing number of studies have detected *S. anginosus* in the urinary tract (21, 173–176). Isolates of *S. anginosus* have been identified in urine samples from individuals experiencing various lower urinary tract symptoms; SAG members, particularly *S. anginosus* are especially enriched in the bladder urine of adult females diagnosed with UUI relative to asymptomatic controls (49% versus 7%) (**Table 1**) (21).

Genetic sequencing of urinary isolates suggests that they belong to a niche-specific clade that may have implications in disease (176). An extensive list of virulence factors has been annotated in these species; however, their role in establishing opportunistic infections is still unclear (177). Antibiotic resistance towards macrolides, aminoglycosides, sulfonamides, and tetracyclines has been observed (178–181) but the resistance profile of urinary isolates has yet to be reported.

### The family *Actinomycetaceae*

Members of the family *Actinomycetaceae* are a phylogenetically diverse group of Gram-positive, facultatively anaerobic or micro-aerophilic, branching rod-shaped bacteria. They are part of the flora of the oropharyngeal, gastrointestinal, and genitourinary tracts of humans and many animals (182). First identified in 1896 with *A. israelii*, these rod-shaped bacilli form colonies with fungus-like branched networks of hyphae, a characteristic that led to the initially incorrect assumption that they were fungi. In most healthy individuals, these organisms are commensal in the mucosal epithelia of hollow organs. However, upon trauma or disruption of the epithelial barrier, access to underlying tissues can lead to actinomycoses characterized by a chronic, granulomatous infectious disease (183–185).

### Actinomyces

Members of the genus *Actinomyces* are Gram-positive, pleomorphic, facultative anaerobic rods that exhibit some branching (182, 186). *Actinomyces* species have been identified in catheterized bladder urine of asymptomatic adult females) but are considerably more prevalent in those with UUI (5% and 23%, respectively) (**Table 1**). Thus, under certain conditions, these organisms could be opportunistic pathogens (187). For example, initially isolated from urine and vaginal secretions, *A. urogenitalis* has been reported in infections associated with long-term use of an intrauterine device (183, 185, 188), in a case of bacteremia following *in vitro* fertilization (189), and in an instance of bacteremia associated with prolonged urinary retention (190). With case reports making up the majority of recorded instances of these organisms, it is clear that studies are needed to determine disease risk factors and antibiotic resistances of infections.

### *Actinomyces*-like organisms

The phylogenetic diversity of this family in combination with new modern diagnostic techniques such as 16S rRNA gene sequencing and MALDI-TOF have led to multiple taxonomic revisions and the introduction of many novel species termed *Actinomyces*-like organisms (ALOs). These include *Actinotignum, Gleimia, Schaallia, Trueperella, Varibaculum*, and *Winkia* (182, 191).

#### Actinotignum.

This genus of facultatively anaerobic Gram-positive rods consists of 4 species: *Actinotignum schaalii, Actinotignum urinale*, *Actinotignum sanguinis*, and *Actinotignum timonense* (192–195). *A. schaalii* and *A. urinale* were first described under the basonyms *Actinobaculum schaalii* and *Actinobaculum urinale*, respectively (192, 193). However, based on the 16S rRNA gene sequence, a remote relationship with the *Actinobaculum suis* type strain was found (Soltys 50052), resulting in reclassification to the *Actinotignum* genus (194, 196).

Although first isolated from blood, both *A. schaalii, A. sanguinis*, and *A. urinale* have since been isolated from urine. *A. schaalii* has most often been reported in the context of UTI (197). For example, it is reported to be an emerging uropathogen of elderly people suffering from UTI with comorbidities (198, 199). It also has been detected in children with urinary tract disorders (200, 201). The genus as a whole has been reported to be significantly more common in adult women with UUI than in unaffected controls (9, 21) (**Table 1**), but the species *A. schaalii* specifically has been found at significantly higher mean abundances in adult women with UUI compared to unaffected controls (202).

Other species have been associated with infections. *A. urinale* was first isolated from human urine of patients with UTI (194); however, it also has been isolated from human blood cultures (203). *A. sanguinis* was first isolated from a human blood culture of a patient with septicemia and has been co-isolated with *Trueperella bernardiae* from breast abscesses in women (204). The first report of *A. timonense* concerned an isolate from the urine of a 59-year-old man with end-stage renal disease (195).

Like many of the species reviewed here, *Actinotignum* species grow slowly under ambient atmospheric conditions typically used by clinical microbiology laboratories; thus, they are often overgrown by faster-growing bacteria. Furthermore, because these species resemble commensal skin and mucosal species, they are often mistakenly identified as contaminants (205). Also, until recently, *Actinotignum* species were difficult to identify after cultivation. However, the advent of molecular techniques has resulted in increasing reports of *A. schaalii* in the context of human infection (198, 199). Further research is essential to determine whether these *Actinotignum* species are uropathogens.

#### Gleimia.

Formerly belonging to the *Actinomyces* genus, the new *Gleimia* genus consists of three members: *G. europea, G. hominis*, and *G. coleocanis*. The first two have been isolated in humans and the latter in dogs. Human isolates have been implicated in UTIs (182, 206) and have been suggested as a bladder cancer marker (207). They can present clinically with persistent ear infections and recurrent soft tissue infections (208–211), as well as abscesses of the neck, back, feet, brain, and genital area in both men and women of various ages (182, 185, 187, 206, 208, 212–214). Recent cases have linked *G. europaea* with necrotizing fasciitis (210, 211) with a recent case report of rapid infection progression and Fournier’s Gangrene (215). Due to ineffective identification techniques, taxon reclassification, and inadequate research, *Gleimia* species remain misunderstood with few reports concerning their pathophysiology.

#### Schaalia.

Like *Gleimia*, a former member of the *Actinomyces* genus, *S. turicensis* and *S. radingae* are Gram-positive, catalase- and urease-negative, anaerobic, filamentous bacilli (182, 216) that have both been isolated from catheterized urine. Originally isolated from a perianal abscess, they were originally classed as CDC Coryneform Group E, from which *A. radingae* and *A. turicensis* were purified and characterized (216). After reassessment of phylogenetic positioning and chemotaxonomic characteristics, these species were reassigned to *Schaalia* along with eleven other species (191), including *S. meyeri* and *S. odontolytica*, all part of the human commensal urinary bladder microbiome.

*S. turicensis* is a commensal of the skin, gut, oral cavity, and female urogenital tract (182) but is also an opportunistic pathogen. Clinical isolates have been reported in bacteremia (217), purent mastoiditis and meningitis (218), infection following rotator cuff repair (219), gonococcal urethritis (220), and a perianal abscess (216). The circumstances and conditions that transform *S. turicensis* from commensal to pathogen remain to be elucidated.

#### Trueperella.

*T. bernardiae* is an emerging opportunistic pathogen in both humans (204, 221–229) and animals (230, 231). In the 1980s, isolates recovered from blood cultures, wounds, abscesses, and skin infections were found to be similar by biochemical testing and described using the provisional name CDC fermentative Coryneform group 2 (CDC group 2) (230). CDC group 2 was formally assigned to the species *Actinomyces bernardiae* based on 16S rRNA gene sequencing and other features for strains recovered from infections in the United States, Canada, and Switzerland (226). In 1997, following reanalysis of the 16S rRNA gene sequences and after comparison with species in the genus *Actinomyces*, *A. bernardiae* was assigned to the genus *Arcanobacterium* (232). After reassessment of phylogenetic positioning and chemotaxonomic characteristics, this species was reassigned to *Trueperella*, along with *A. abortisuis, A. bialowiezensis, A. bonasi* and *A. pyogenes* (233). Of these five organisms, only *T. bernardiae* and *T. pyrogenes* have been reported in humans, all associated with mild to severe infections and abscesses. As all 5 species have been reported as pathogens in animals, it has yet to be established if *T. bernardiae* and *T. pyrogenes* are commensals in skin, oropharynx, and urinary tract or are opportunistic zoonotic pathogens of humans (225, 231, 233). The occurrence of *T. bernardiae* in polymicrobial infections may reflect dependence of this organism on nutrients provided by other species. Immunosuppressed patients appear to be more at risk for infection by *T. bernardiae* (94).

#### Varibaculum.

The first member of this anaerobic, diphtheroid, Gram-positive genus was initially characterized as a distinct species with resemblance to the genus *Actinomyces* in 2003 (234). Case reports have associated *V. cambriensis* in polymicrobial, anaerobic human abscess infections (235). The source of these infections remains unknown, and it is unclear if this microbe depends on one or more partner species for survival or infection. Before the advent of MALDI-TOF MS, accurate identification of *V. cambriense* in routine clinical microbiology laboratories was difficult (233). Thus, in the past, this species may have been dismissed as contamination (97). Indeed, the use of more modern detection methods have identified members of the genus *Varibaculum* in human urine, as well as prostate and bladder cancer (236–238). Whereas metaculturomic methods rarely detect this anaerobe, it is frequently detected by metagenomic approaches. How *Varibaculum* species cause disease remains poorly understood.

#### Winkia.

*Actinomyces neuii* was discovered in 1994 (239). Recently, it was given its own genus *Winkia* (191). This catalase-positive coccobacillus has been found in asymptomatic women (240) but may be an opportunistic emerging pathogen in humans. Infections include abscesses and infected atheromas (241), cellulitis (242), endophthalmitis, and UTIs (185), as well as bacteremia, including endocarditis (243). Isolates have also been implicated in neonatal sepsis (244–246) and bacterial vaginosis (247). In all cases, how *W. neuii* is mechanistically involved in these diseases is poorly described. Antibiotic resistance to fluoroquinolones has been observed (248), but wider studies into urinary isolate resistances are needed. Like other Gram-positive rods, it is often dismissed as a contaminant (249). We have found it to be highly enriched in adult females with UUI relative to asymptomatic controls (28% and 3%, respectively) (21) (**Table 1**).

### The genus *Corynebacterium*

The Gram-positive genus *Corynebacterium* includes approximately 80 recognized species. These are catalase-positive rods with occasional swelling or club-like ends. The envelopes of most but not all contain mycolic acid. Nine species are lipophilic (able to metabolize lipids), asaccharolytic (unable to metabolize carbohydrates), and strictly aerobic. The rest are non-lipophilic and saccharolytic; some of these are fermentative facultative anaerobes, while others are non-fermentative aerobes (250).

Although *Corynebacterium* species are typically commensals of the mucous membranes of hollow organs and skin, some are opportunistic pathogens. Many species have been isolated from the bladder urine of asymptomatic adult females (50) but the genus is particularly enriched in those diagnosed with UUI (8% and 52%, respectively (21), (**Table 1**). Seven species that occur often and either are or could be urinary tract opportunists are summarized in **Table 2** (250). Here, we review 2 of them: *C. amycolatum* and *C. urealyticum.*

**Table 2:** Selected bladder urine commensal *Corynebacteria* reported as opportunistic pathogens.

Organism	Characteristics	Urease	Clinical Conditions/Isolates	References
*C. amycolatum*	Non-lipophile, Aerobe	+	Blood culture, cellulitis, endocarditis, mastitis, peritonitis, sepsis, wounds	(250–253)
*C. aurimucosum*	Non-lipophile, Facultative anaerobe	–	Blood culture, complications of pregnancy, UTI	(249, 250, 254)
*C. glucuronolyticum *	Non-lipophile, Facultative anaerobe	+	Chronic prostatitis, cystitis, infertility, persistent urethritis	(250, 255, 256)
*C. minutissimum *	Non-lipophile, Aerobe, Facultative anaerobe	+	Bacteremia, meningitis, endocarditis, cellulitis, abscesses, peritonitis, pyelonephritis	(250, 252)
*C. riegelii*	Non-lipophile, Facultative anaerobe	+	Blood cultures, urosepsis, UTI	(250, 257, 258)
*C. tuberculostearicum*	Lipophile, Facultative anaerobe	–	Abscesses, blood culture, mastitis, peritonitis	(250, 252, 259)
*C. urealyticum*	Lipophile, Microaerophile	+	Acute cystitis, alkaline encrusted cystitis, encrusted pyelitis, endocarditis, kidney and bladder stones, pyelonephritis, UTI	(250, 260, 261)

#### C. amycolatum

is a facultative anaerobic fermenter that is non-lipophilic (250, 251). It is unusual, as it lacks mycolic acid, which is common in other coryneforms. Although a commensal of skin and mucous membranes, *C. amycolatum* can be an opportunistic pathogen, especially in immunosuppressed patents and nosocomial environments. It has been isolated from blood cultures, cellulitis, wounds, endocarditis, and peritonitis (250, 253). A recent pan-genomic study of drug resistant and commensal isolates of *C. amycolatum* gave insight into the core genome and the transition from commensal to pathogenic phenotype (262).

#### C. urealyticum

is an asaccharolytic, lipophilic coryneform that expresses lipase and strong urease activity (250, 261, 263). *C. urealyticum* is an opportunistic nosocomial pathogen that can cause acute cystitis, pyelonephritis, alkaline-encrusted cystitis, and encrusted pyelitis (250). It has been associated with bacteremia, mainly in patients with chronic urological diseases and its strong urease activity is a major factor in urinary stone formation (260). Painful and persistent pathologies occur associated with encrustations in the kidney, ureters, and urethra due to alkalinization of urine from metabolism of urea (261). It tends to be multi-drug resistant. In a study of *C. urealyticum* infections in kidney transplant recipients, between 40 to 85% of the isolates tested were resistant to azithromycin, cefotaxime, chloramphenicol, ciprofloxacin, clindamycin, erythromycin, gentamycin, norfloxacin, penicillin G, or tetracycline (261) One non-antibiotic treatment relies on oral L-methionine, which when metabolized acidifies urine (264, 265). It is unclear if this treatment is bactericidal or bacteriostatic, as in the original report removing methionine resulted in return of the uropathogens (264). *C. urealyticum* is also a zoonotic pathogen associated with UTIs in dogs, cats and other animals (250).

### The order *Micrococcales*

The order *Micrococcales* is comprised of eighteen families including *Dermabacteraceae* and *Micrococcaceae* (191), comprised of 3 and 19 genera, respectively. Several of these genera are detected in human bladder urine including *Dermabacter, Kokura*, *Pseudoglutamicibacter*, and *Rothia* (**Table 1**). Here, we review *D. hominis* and both *Pseudoglutamicibacter albus* and *P. cumminsii.*

#### *Dermabacter*.

The *Dermabacter* genus contains three species: *D. hominis, D. jinjuensis*, and *D. vaginalis*, of which only *D. hominis* has been found in human catheterized urine (**Table 1**). First described in 1988 (266), this Gram-positive, non-spore forming, non-acid fast, facultative anaerobic short rod is considered to be a commensal of human skin (267). However, *D. hominis* has been reported in diverse clinically relevant scenarios, almost always as part of polymicrobial communities in patients that are immunocompromised or suffering with significant comorbidities, most often cardiovascular disease, diabetes mellitus, and chronic kidney disease (267). Other reports exist of its isolation from biopsies of bone and joint infections and swabs of soft tissue infection (267), a case of trichobacteriosis axillaris (268), a neck sebaceous cyst (269), blood cultures of patients with bacteremia (270), peritoneal fluid from a patient with end stage renal disease (271), recurrent abscesses (272), bone deposits from a patient with chronic osteomyelitis (273), breast implant infections (274), and cerebral abscess of a renal transplant patient (275). In one report, *D. hominis* isolated from human semen was found to be capable of forming a strong biofilm, which could potentially be a cause of prostatitis (276). Thus, in immunocompromised patients or those with comorbidities, *D. hominis* may be pathogenic. Although we have detected it in the bladder urine of adult females with UTI and UUI (**Table 1**), the relationship between this microbe and the urinary tract remains unclear.

#### Pseudoglutamicibacter.

Originally assigned to Centers for Disease Control and Prevention coryneform group B-1 and B-3 (277) and later the genus *Arthrobacter* (278), the recently established genus *Pseudoglutamicibacter* contains two species: *P. cumminsii* and *P. albus* (279). It is unknown whether these species are commensal microbes or opportunistic pathogens, and we have detected both species in the bladder urine of both asymptomatic and affected adult females (1% and 11%, respectively) (**Table 1**). However, most samples collected from humans have been associated with severe infections and abscesses, including infected amniotic fluid, chronic cervicitis, chronic otorrhea, external otitis, calcaneus osteomyelitis, sepsis, and UTI (277, 280). Isolation sites have included blood, bone, amniotic fluid, leg wounds, and urine (277, 280).

Both *P. cumminsii* and *P. albus* are Gram-positive, mesophilic, catalase-positive, obligate aerobe coccobacilli (278, 279). *P. cumminisii* is the most frequently encountered member of the genus in human clinical specimens (280). A recent case study identified *P. cumminsii* in the urine culture of a woman with UTI (281). The first clinical specimen of *P. albus* was isolated from a blood culture of a surgical patient with severe phlebitis (278). The 16S rRNA genes of *P. cumminsii* and *P. albus* share a high degree of homology. This makes distinguishing these two organisms difficult (279). Better differentiation will require whole genome sequencing of isolates and better defined MALDI-TOF profiles.

### The family *Bifidobacteriaceae*

The family *Bifidobacteriales* consist of 5 *genera: Bifidobacterium*, *Gardnerella*, *Alloscardovia*, *Scardovia* and *Parascardovia* (191). Of these, *Bifidobacterium*, *Gardnerella* and *Alloscardovia* have been detected in human bladder urine (**Table 1**). While the role of *Bifidobacterium* in colonizing the gastrointestinal tract is well known (282), its role in the urinary tract remains undefined. *Gardnerella* species are prevalent and abundant in the urinary tracts of asymptomatic adult females but are somewhat more prevalent in women with UUI (13% and 21%, respectively (9, 10) (**Table 1**). The role of *G. vaginalis* in bacterial vaginosis and a link to UTI have been observed, but the mechanisms remain elusive (82). Below, we review a lesser-known member of this family *Alloscardovia omnicolens*.

#### *Alloscardovia*.

Several species belong to the genus *Alloscardovia*, but only *A. omnicolens* has been described in human urine, especially from UUI patients (**Table 1**). It is a Gram-positive, oxidase and catalase-negative, non-spore-forming, anaerobic rod (283, 284). While originally considered to be a commensal of the gastrointestinal tract and oral cavity, there is evidence that this microbe is clinically significant and should not be ignored if found in clinical specimens, especially if isolated from the urinary tract (285). The *Alloscardovia* genus was first described in 2007 by Huys and co-authors after sampling various clinical sites, including the urethra, urine, blood, abscesses of the lung and aorta, and tonsils (286). *A. omnicolen*s has been identified in urine cultures of bladder cancer patients with concurrent UTI (285). It also has been considered the probable cause of infection for at least one UTI (283) and a case of bacteremia due to a UTI in a 70-year-old woman with advanced uterine cancer (287). Therefore, *A. omnicolens* appears to be an opportunistic pathogen. Antibiotic resistance to metronidazole and moxifloxacin has been described (288, 289).

### The order *Eubacteriales*

The order *Eubacteriales* is comprised of at least 25 families, all anaerobes. Of these families, members of *Clostridiaceae* (290, 291) and *Peptostreptococcacae* (40, 292) are reviewed here. Anaerobes are not detected by SUC (27). Oxygen toxicity complicates the collection and culturing these microbes (28, 30), making it difficult to obtain sufficient material for characterization (27, 293). However, with the advent of molecular diagnostic techniques and enhanced culture methods, the role of anaerobes in both the commensal flora and as opportunistic pathogens is becoming recognized (27).

#### Thomasclavelia ramosum

Is a Gram-positive obligate anaerobic bacillus with the ability to hydrolyze esculin (290, 293–297). As such, it is rarely cultured, even by EQUC, but it is observed by metagenomic approaches.

Discovered in 1898, it was named *Bacillus ramosum* then renamed *Ramibacterium ramosum* (295, 297). The demonstration of sporulation led to its reclassification as *Clostridium ramosum* (294, 297). Further dissections of the genus *Clostridium*, using a combination of genetic markers, led to another name change, this time to *Erysipelatoclostridium ramosum* and most recently to *Thomasclavelia ramosum* (290, 293, 296).

While found as part of the commensal flora in the gastrointestinal and urinary tracts, this organism has been documented in infections, such as appendicitis, blood, brain abscess, bacteremia, joint infections, and pulmonary gangrene (295, 297, 298). It also is one of the few sporulating bacteria detected in the urinary microbiome. Further study is warranted to understand how this commensal becomes an opportunistic and potent pathogen.

#### Peptostreptococcus anaerobius.

Originally described in 1936, the genus *Peptostreptococcus* consists of four species, *P. anaerobius, P. canis, P. russellii*, and *P. stomatis* (40, 292, 299). These are Gram-positive anaerobic cocci; thus, they are rarely cultured but are frequently detected by metagenomic approaches. They have weak fermentative and proteolytic metabolisms. Consequently, it may be symbiotic with other organisms from which it derives nutrients (300). Indeed, *P. anaerobius* has been isolated most often from polymicrobial infections of soft tissue, bone, brain, implant-related and respiratory tract infections (40, 292, 299). *P. anaerobius* is also one of the most common Gram-positive, anaerobic cocci isolated from infections of the female urogenital tract and the abdominal cavity (292). Thus, while *P. anaerobius* is probably a commensal of the gastrointestinal, vaginal, and urinary tracts, it can be an opportunistic pathogen, particularly within polymicrobial infections.

### The order *Bacteroidales*

The order *Bacteroidales* is comprised of 17 families. The best known are *Bacteroidaceae* and *Prevotellaceae.* The latter family consists of Gram-negative anaerobes split into four genera: *Hallella*, *Paraprevotella*, *Prevotella*, and *Alloprevotella* (301). Below, we will review a few species.

#### Prevotella.

The genus *Prevotella* consists of 55 distinct species of Gram-negative coccobacilli anaerobes that are commonly found in the oral, gastrointestinal, and urogenital tracts of humans and animals. It was originally proposed to characterize the moderately saccharolytic, oral *Bacteroide*s species (302). Until recently, the lack of characteristic phenotypic and biochemical traits had hampered identification at the species level among this group of obligatory anaerobes. They are rarely cultured, but the availability of 16S rRNA sequence analysis has improved detection, and thus the number of recognized *Prevotella* species has increased over the last few years (303). Recently, a genomic and functional analysis of the 55 phenotypically, ecologically and functionally diverse species comprising *Prevotella* identified 7 distinct clades and thus reassignment across 7 genera, with 4 of them being new genera: *Segatella*, *Hoylesella*, *Leyella* and *Palleniella* (304). Below, we review *Prevotella bivia* and *Hoylesella timonensis.*

#### *Prevotella bivia*.

Previously classified as *Bacteroides bivius*, *P. bivia* is commonly found in the human vaginal microbiome (305). Though normally a commensal, *P. bivia* has pathogenic potential to trigger severe infection and induce tissue destruction, especially when there is excess estrogen and with the synergistic cooperation of other species (306). *P. bivia* is one of the most frequently isolated anaerobic bacteria in cases of bacterial vaginosis. The presence of *P. bivia* creates an environment that facilitates growth of *Peptostreptococcus anerobius* (300) and *Gardnerella vaginalis* (307). *P. anaerobius* growth is enhanced with production of certain amino acids by *P. bivia*. Likewise, *G. vaginalis* growth is enhanced with production of ammonia by *P. bivia*.

*P. bivia* is one of the most frequently isolated bacteria in women with pelvic inflammatory disease, as noted in a retrospective, cross-sectional study (308), It has also been implicated in cases of recurrent UTIs, osteomyelitis (309), osteitis (310), endocarditis (311), necrobacillosis (312), sinusitis (313), wound infections from animal bites (314, 315), intracranial abscesses (316), periodontal and tubo-ovarian abscesses (317), and adverse pregnancy outcomes such as preterm labor (318). The virulence factors of *P. bivia* are not fully understood, but research suggests that they may include adhesins that allow the bacteria to attach to host cells, enzymes that degrade host tissue, and toxins that damage host cells and stimulate inflammation. As with other members of the genus *Prevotella*, antibiotic resistance is becoming an increasing concern; the most common are amoxicillin-clavinate, clindamycin, and moxifloxacin (319). Thus, further research into *P. bivia* is warranted.

#### Hoylesella timonensis.

*Prevotella timonensis* was first isolated from a human breast abscess (320). After reassessment of phylogenetic positioning and chemotaxonomic characteristics, this species was reassigned to *Hoylesella* (304). Like the previously described anaerobes, *H. timonensis* is rarely cultured but detected often by metagenomics. The breast abscess isolate mentioned above ferments glucose, maltose, and lactose. It also hydrolyzes esculin but is urease and catalase negative. Although the species has most often been isolated from cutaneous/soft tissue abscesses and bone infections, it is also prevalent in human genitourinary samples, including from urine (304, 321). While *H. timonensis* may be a commensal organism in the genitourinary tract, it has been associated with bacterial vaginosis (322). Thus, some evidence supports its role as an emerging opportunistic pathogen (321, 322).

## Concluding remarks

This review provides a robust description of lesser-known microbes to support our recommendation that our understanding of uropathogens should go beyond the “usual suspects.” Current clinical diagnosis is hampered by limitations in what clinical microbiologists detect in culture in as little incubation time as possible, most often under atmospheric oxygen conditions (323). This current approach underreports fastidious, slow growing, and anaerobic bacteria, many of which are generally excluded as uropathogens despite evidence to the contrary (12, 27, 292). The clinical consequences include the common scenario of repeated negative standard urine culture results despite relevant, persistent symptoms (102). Common sense should lead one to suspect that some undetected agent(s) play(s) a role in these persistent symptoms and the most likely candidates are fastidious, slow growing and/or anaerobic bacteria. The standard method dismisses, underreports, or does not detect these microbes but they are repeatedly detected by more sensitive metaculturomic (enhanced culture-dependent) or metagenomic (culture-independent, DNA-based methods) (324).

None of the reviewed species were discovered recently. For example, *Thomasaclava* (formerly *Clostridium*) *ramnosum* dates back more than a century, while others (e.g., *A. urinae*) were discovered at least 10 years ago. Yet, little is known of their pathophysiology. Even their phylogeny is poorly understood, as highlighted by the plethora of name changes (191, 293, 304). As microbial detection technologies have improved, especially with the advent of MALDI-TOF MS, so too have the detection and reporting of these microbes. However, description of these microbes in relation to disease should not be confined to sporadic case reports as has been the case so far.

This review is a call to action to fill this knowledge gap, to begin studies designed to determine first their functioning as commensals and then their transition to opportunistic pathogens. Both retrospective and prospective studies are greatly needed to determine risk factors for symptomatic infections, especially for microbes that have the ability to cause severe complications that might be entirely preventable with proper and early diagnosis. The global rise in antibiotic resistance is well established but only for microbes under active surveillance. Until the resistance profiles of urinary isolates are better reported, patients will continue to experience therapy failures. Thus, as long as we remain blind to the activities and capabilities of these emerging uropathogens, preventable damage will continue to afflict patients and will most definitely worsen as these microbes continue to evolve.

## Author contributions

AW conceived of the manuscript. All authors contributed to the article and approved the submitted version.
