# Supplementary material for: Beyond the usual suspects: emerging uropathogens in the microbiome age
Source: Front Urol. 2023 Jul 26;3:1212590. doi: 10.3389/fruro.2023.1212590 (PMC12327349; doi:10.3389/fruro.2023.1212590)
Supplement: Supplementary file 1 [file DataSheet_1.docx]

**Appendix 1. Methods for Beyond the Usual Suspects Table 1**

The frequency table (Table 1) was generated from studies assessing the urobiomes of patients with and without lower urinary tract symptoms. Species frequency is organized by patient group: UUI (n=253), UTI (n=304), SUI (n=50), IC/PBS (n=49), and Control (n=351). Total n=1007. Species were isolated via EQUC or SUC and identified via MALDI-TOF MS. Redundancies of species from the same patient (due to longitudinal or duplicate sampling) were omitted.

Data were pulled from the following previously published studies:

| UTI-EQUC | <https://pubmed.ncbi.nlm.nih.gov/26962083/> |
| --- | --- |
| MIR | <https://pubmed.ncbi.nlm.nih.gov/35412069/> |
| SVE | <https://pubmed.ncbi.nlm.nih.gov/34184930/> |
| EST/Estrogen | <https://pubmed.ncbi.nlm.nih.gov/32791124/> |
| Controls study (R01) | <https://pubmed.ncbi.nlm.nih.gov/31469215/> |
| ICFUM | <https://pubmed.ncbi.nlm.nih.gov/32265402/> |
| SOL | <https://pubmed.ncbi.nlm.nih.gov/36422657/> |
| UvS | <https://pubmed.ncbi.nlm.nih.gov/26423260/> |

IRB approvals: LU207102, LU207152, LU207777, LU209545, LU202567, LU204195, LU204133, LU206499, LU204658

Clinical Trials: NCT03190421, NCT02524769, NCT02835846, NCT01642277, NCT02495389
