# Supplementary material for: Beyond the usual suspects: emerging uropathogens in the microbiome age
Source: Front Urol. 2023 Jul 26;3:1212590. doi: 10.3389/fruro.2023.1212590 (PMC12327349; doi:10.3389/fruro.2023.1212590)
Supplement: Supplementary file 2 [file DataSheet_2.docx]

| **Organism** | **Bacterial Diversity Database Type Stain Link** | **NCBI Taxonomy ID^1^** | **Reference** |
| --- | --- | --- | --- |
| *Actinotignum* *sanguinis* | <https://bacdive.dsmz.de/strain/130140> | 1445614 | Yassin et al. 2015 |
| *Actinotignum schaalii,* | <https://bacdive.dsmz.de/strain/145> | 59505 | Price et al., 2016, Yassin et al., 2015 |
| *Actinotignum* *timonense* | Not available | 1870995 | Brahimi et al., 2017 |
| *Actinotignum* *urinale,* | <https://bacdive.dsmz.de/strain/146> | 190146 | Hall et al., 2003a |
| *Alloscardovia omnicolens* | <https://bacdive.dsmz.de/strain/1767> | 419015 | Price et al., 2016 |
| *Dermabacter hominis* | <https://bacdive.dsmz.de/strain/3918> | 36740 | Jones and Collins, 1988 |
| *Facklamia hominis* | <https://bacdive.dsmz.de/strain/138280> | 178214 | Collins et al., 1997 |
| *Gleimia europaea* | <https://bacdive.dsmz.de/strain/138197> | 66228 | Funke et al., 1997 |
| *Glemia hominis* | <https://bacdive.dsmz.de/strain/194> | 595468 | Funke et al., 2010 |
| *Globicatella saunguinis* | <https://bacdive.dsmz.de/strain/231> | 13076 | Collins et al., 1992 |
| *Nosocomiicoccus massiliensis* | <https://bacdive.dsmz.de/strain/24598> | 1232430 | Mishra et al., 2013 |
| *Peptostreptococcus anaerobius* | <https://bacdive.dsmz.de/strain/11843> | 1261 | Legaria et al., 2021 |
| *Prevotella bivia* | <https://bacdive.dsmz.de/strain/12525> | 28125 | Snydman et al., 1980 |
| *Pseudoglutamicribacter albus* | <https://bacdive.dsmz.de/strain/7583> | 98671 | Busse 2016; Wauters et al., 2000 |
| *Pseudoglutamicribacter cumminsi* | <https://bacdive.dsmz.de/strain/7578> | 156979 | Busse 2016; Funke et al., 1996 |
| *Thomasclavelia ramosum* | <https://bacdive.dsmz.de/strain/2653> | 1547 | Legaria et al., 2020 |
| *Trueperella bernardiae* | <https://bacdive.dsmz.de/strain/214> | 59561 | Yassin et al., 2011 |
| *Varibaculum cambriense* | <https://bacdive.dsmz.de/strain/210> | 184870 | Hall et al., 2003b |
| *Winkia neuii* | <https://bacdive.dsmz.de/strain/166> | 33007 | Funke et al., 1994 |

^1^NCBI Taxonomy Database Link: <https://www.ncbi.nlm.nih.gov/Taxonomy/Browser/wwwtax.cgi>

**Appendix 2. Emerging Uropathogen Resources**

It should be noted that the type strains in the Bacterial Diversity Database are not urinary bladder specific. In contrast, species described in Table 1 were isolated from bladder urine obtained from female patients by transurethral catheterization. The information here is to provide strain information and growth conditions. Bladder urine-derived species will be reported in submitted and future publications and information/strains can be obtained by emailing the corresponding author ([awolfe@luc.edu](mailto:awolfe@luc.edu)).

**REFERENCES**

Brahimi S, Cadoret F, Fournier PE, Moal V, Raoult D. (2017) *'Actinotignum timonense'* sp. nov., a new bacterial species isolated from a human urine sample. *New Microbes New Infect*. **16:**47-48.

doi: 10.1016/j.nmni.2017.01.002.

<https://pubmed.ncbi.nlm.nih.gov/28203375/>

Busse HJ. (2016) Review of the taxonomy of the genus *Arthrobacter*, emendation of the genus *Arthrobacter sensu lato*, proposal to reclassify selected species of the genus *Arthrobacter* in the novel genera *Glutamicibacter* gen. nov., *Paeniglutamicibacter* gen. nov., *Pseudoglutamicibacter* gen. nov., *Paenarthrobacter* gen. nov. and *Pseudarthrobacter* gen. nov., and emended description of *Arthrobacter roseus*. *Int J Syst Evol Microbiol.* **66(1):**9-37.

doi: 10.1099/ijsem.0.000702.

<https://pubmed.ncbi.nlm.nih.gov/26486726/>

Collins MD, Aguirre M, Facklam RR, Shallcross J, Williams AM. (1992) *Globicatella sanguis* gen.nov., sp.nov., a new gram-positive catalase-negative bacterium from human sources.

*J Appl Bacteriol.* **73(5):**433-7.

doi: 10.1111/j.1365-2672.1992.tb05000.x.

<https://pubmed.ncbi.nlm.nih.gov/1280253/>

Collins MD, Falsen E, Lemozy J, Akervall E, Sjödén B, Lawson PA. (1997) Phenotypic and phylogenetic characterization of some *Globicatella*-like organisms from human sources: description of *Facklamia hominis* gen. nov., sp. nov. *Int J Syst Bacteriol*. **47(3):**880-2.

doi: 10.1099/00207713-47-3-880.

<https://pubmed.ncbi.nlm.nih.gov/9226922/>

.

Funke G, Alvarez N, Pascual C, Falsen E, Akervall E, Sabbe L, Schouls L, Weiss N, Collins MD. (1997) *Actinomyces europaeus* sp. nov., isolated from human clinical specimens *Int J Syst Bacteriol*. **47(3):**687-92.

doi: 10.1099/00207713-47-3-687.

<https://pubmed.ncbi.nlm.nih.gov/9226901/>

Funke G, Englert R, Frodl R, Bernard KA, Stenger S. (2010) *Actinomyces hominis* sp. nov., isolated from a wound swab. *Int J Syst Evol Microbiol.* **60(Pt 7):**1678-1681.doi: 10.1099/ijs.0.015818-0.

<https://pubmed.ncbi.nlm.nih.gov/19734287/>

Funke G, Hutson RA, Bernard KA, Pfyffer GE, Wauters G, Collins MD. (1996) Isolation of *Arthrobacter* spp. from clinical specimens and description of *Arthrobacter cumminsii* sp. nov. and *Arthrobacter woluwensis* sp. nov. *J Clin Microbiol*. 1996 Oct;34(10):2356-63.

doi: 10.1128/jcm.34.10.2356-2363.1996.

<https://pubmed.ncbi.nlm.nih.gov/8880479/>

Funke G, Stubbs S, von Graevenitz A, Collins MD. (1994) Assignment of human-derived CDC group 1 coryneform bacteria and CDC group 1-like coryneform bacteria to the genus *Actinomyces* as *Actinomyces neuii* subsp. *neuii* sp. nov., subsp. nov., and *Actinomyces neuii* subsp. anitratus subsp. nov. *Int J Syst Bacteriol*. 1994 Jan;44(1):167-71.

doi: 10.1099/00207713-44-1-167.

<https://pubmed.ncbi.nlm.nih.gov/8123558/>

Hall V, Collins MD, Hutson RA, Falsen E, Inganäs E, Duerden BI. (2003a) *Actinobaculum urinale* sp. nov., from human urine *Int J Syst Evol Microbiol*. **53(Pt 3):**679-682.

doi: 10.1099/ijs.0.02422-0.

<https://pubmed.ncbi.nlm.nih.gov/12807186/>

Hall, V., M. D. Collins, P. A. Lawson, R. A. Hutson, E. Falsen, E. Inganas, and B. Duerden. (2003b). 'Characterization of some actinomyces-like isolates from human clinical sources: description of *Varibaculum* *cambriensis* gen nov, sp nov', *J Clin Microbiol*, 41: 640-4.

doi: 10.1128/JCM.41.2.640-644.2003.

<https://pubmed.ncbi.nlm.nih.gov/12574260/>

Jones D. and Collins MD. (1988). Taxonomic studies on some human cutaneous coryneform bacteria: Description of *Dermabacter hominis* gen. nov., sp. nov. FEMS Microbiology Letters, **51(1),** 51-55.

doi: 10.1111/j.1574-6968.1988.tb02967.x

<https://academic.oup.com/femsle/article/51/1/51/489756>

Legaria MC, García SD, Tudanca V, Barberis C, Cipolla L, Cornet L, Famiglietti AMR, Stecher D, Vay CA. (2020) *Clostridium ramosum* rapidly identified by MALDI-TOF MS. A rare gram-variable agent of bacteraemia. *Access Microbiol.* **2(8):**acmi000137.

doi: 10.1099/acmi.0.000137.

<https://pubmed.ncbi.nlm.nih.gov/32974599/>

Legaria MC, Nastro M, Camporro J, Heger F, Barberis C, Stecher D, Rodriguez CH, Vay CA. (2021) *Peptostreptococcus anaerobius*: Pathogenicity, identification, and antimicrobial susceptibility. Review of monobacterial infections and addition of a case of urinary tract infection directly identified from a urine sample by MALDI-TOF MS. *Anaerobe*. **72:**102461.

doi: 10.1016/j.anaerobe.2021.102461

<https://pubmed.ncbi.nlm.nih.gov/34626800/>

Mishra AK, Edouard S, Dangui NP, Lagier JC, Caputo A, Blanc-Tailleur C, Ravaux I, Raoult D, Fournier PE. (2013) Non-contiguous finished genome sequence and description of *Nosocomiicoccus massiliensis* sp. nov. *Stand Genomic Sci.* **9(1):**205-19.

doi: 10.4056/sigs.4378121.

<https://pubmed.ncbi.nlm.nih.gov/24501657/>

Price TK, Dune T, Hilt EE, Thomas-White KJ, Kliethermes S, Brincat C, Brubaker L, Wolfe AJ, Mueller ER, Schreckenberger PC. (2016) The Clinical Urine Culture: Enhanced Techniques Improve Detection of Clinically Relevant Microorganisms. *J Clin Microbiol.* **54(5):**1216-22.

doi: 10.1128/JCM.00044-16.

<https://pubmed.ncbi.nlm.nih.gov/26962083/>

Snydman, D. R., F. P. Tally, R. Knuppel, J. Landrigan, S. L. Gorbach, and J. G. Bartlett. 1980. *'Bacteroides bivius* and *Bacteroides disiens* in obstetrical patients: clinical findings and antimicrobial susceptibilities', *J Antimicrob Chemother*, **6:** 519-25.

doi: 10.1093/jac/6.4.519.

<https://pubmed.ncbi.nlm.nih.gov/6968748/>

Wauters G, Charlier J, Janssens M, Delmée M. (2000) Identification of *Arthrobacter oxydans*, *Arthrobacter luteolus* sp. nov., and *Arthrobacter albus* sp. nov., isolated from human clinical specimens. *J Clin Microbiol.* **38(6):**2412-2415.

doi: 10.1128/JCM.38.6.2412-2415.2000.

<https://pubmed.ncbi.nlm.nih.gov/10835019/>

Yassin AF, Hupfer H, Siering C, Schumann P. (2011) Comparative chemotaxonomic and phylogenetic studies on the genus *Arcanobacterium* Collins et al. 1982 emend. Lehnen et al. 2006: proposal for *Trueperella* gen. nov. and emended description of the genus *Arcanobacterium*. *Int J Syst Evol Microbiol*. **61(Pt 6):**1265-1274.

doi: 10.1099/ijs.0.020032-0.

<https://pubmed.ncbi.nlm.nih.gov/20622055/>

Yassin AF, Spröer C, Pukall R, Sylvester M, Siering C, Schumann P. (2015) Dissection of the genus *Actinobaculum*: Reclassification of *Actinobaculum schaalii* Lawson et al. 1997 and *Actinobaculum urinale* Hall et al. 2003 as *Actinotignum schaalii* gen. nov., comb. nov. and *Actinotignum urinale* comb. nov., description of *Actinotignum sanguinis* sp. nov. and emended descriptions of the genus *Actinobaculum* and *Actinobaculum suis*; and re-examination of the culture deposited as *Actinobaculum massiliense* CCUG 47753T ( = DSM 19118T), revealing that it does not represent a strain of this species. *Int J Syst Evol Microbiol*. 2015 Feb;**65(Pt 2):**615-624.

doi: 10.1099/ijs.0.069294-0.

<https://pubmed.ncbi.nlm.nih.gov/25406238/>
